# Anchorage-dependent multicellular aggregate formation induces a quiescent stem-like intractable phenotype in pancreatic cancer cells

**DOI:** 10.18632/oncotarget.25732

**Published:** 2018-07-06

**Authors:** Yukiko Miyatake, Yusuke Ohta, Shunji Ikeshita, Masanori Kasahara

**Affiliations:** ^1^ Department of Pathology, Faculty of Medicine and Graduate School of Medicine, Hokkaido University, Sapporo, 060-8638, Japan

**Keywords:** pancreatic cancer, collective cell behavior, Ad-MCA, coculture, HEK293T, Pathology

## Abstract

Pancreatic ductal adenocarcinoma (PDAC) is one of the most lethal refractory cancers. Aggressive features in PDAC cells have been well studied, but those exhibited by a population of PDAC cells are largely unknown. We show here that coculture with epithelial-like feeder cells confers more malignant phenotypes upon PDAC cells forming anchorage-dependent multicellular aggregates (Ad-MCAs, a behavior of collective cells), *in vitro*. When CD44v3-10^high^/CD44s^low^ PDAC cell lines, which exhibited an epithelial phenotype before the onset of epithelial–mesenchymal transition (EMT), were cocultured with a monolayer of HEK293T cells overnight, they formed Ad-MCAs on the feeder layer and acquired gemcitabine resistance. CD44v8-10 expression was dramatically increased and Ki-67 staining decreased, suggesting that PDAC cells forming Ad-MCAs acquired cancer stem cell (CSC)-like intractable properties. We found that highly downregulated genes in PDAC cells cocultured with HEK293T cells were significantly upregulated in malignant lesions from pancreatic cancer patients. Our work implies that PDAC cells forming Ad-MCAs partially return to a normal tissue gene profile before the onset of EMT. The collective cell behavior like Ad-MCA formation by PDAC cells may mimic critical events that occur in cancer cells at the very early phase of metastatic colonization.

## INTRODUCTION

Pancreatic ductal adenocarcinoma (PDAC), which constitutes approximately 90% of pancreatic cancers, is one of the most lethal malignant tumors [1]. Once PDACs become detectable, they progress from T1 stage to T4 stage in approximately 14 months [2]. There have been no improvements of survival in patients with advanced pancreatic cancer since the introduction of gemcitabine (GM) [3]. One undesired effect of GM is that it promotes the invasiveness of PDAC cells through reactive oxygen species (ROS)-dependent upregulation of CXCR4 [4]. Accumulating evidence indicates the existence of pancreatic cancer stem cells (CSCs) [5]. A striking clinical feature of CSCs is cell cycle quiescence, which enables them to become resistant to a number of conventional chemotherapeutic agents impairing mitosis such as GM [6]. The properties of CSCs are thought to be regulated by the surrounding microenvironment (niche) [7]. However, the mechanisms by which CSCs emerge and are maintained in the niche are poorly understood because of the difficulty in the experimental expansion of CSCs *in vitro*. Current evidence indicates that CD44, a CSC marker, directly reprograms stem cell properties in colon cancer cells [8].

Epithelial–mesenchymal transition (EMT) generates cells with properties of CSCs [9]. A recent report clearly showed that CD44 splicing isoform-switching from variant isoforms (CD44v) to the standard isoform (CD44s) in tumor epithelium occurs during EMT in breast cancer progression [10]. Notably, a recent study demonstrated that expression of CD44v8-10 contributes to ROS defense by upregulating the synthesis of a major antioxidant, glutathione [11]. Generally, EMT is believed to be one of the essential events for the acquisition of metastatic ability in a variety of carcinomas [9, 12–15]. However, a recent study has reported that carcinoma cells could metastasize without activating EMT programs in genetically engineered mouse models of PDAC development [16, 17].

Previously, we reported that when cocultured with epithelial-like feeder cells, lymphoma cells form anchorage-dependent multicellular aggregates (Ad-MCAs) and that a fraction of aggregate-forming lymphoma cells acquire quiescent CD44-high CSC-like phenotypes. This observation suggests that the intractability of tissue-infiltrating lymphoma cells may be partly accounted for by the acquisition of CSC-like properties [18, 19]. Indeed, recent work indicates that multicellular aggregate (MCA) formation determines gene expression, proliferation, drug resistance, and immune escape *in vitro* in follicular lymphoma [20], suggesting that it affects the malignant potential of the tumor. Metastatic carcinoma cells also occur as both single cells and MCAs in several solid tumors, pointing to the importance of MCA formation [21, 22]. However, the mechanism underlying collective cell behavior such as MCA formation and Ad-MCA formation is poorly understood.

In this study, we demonstrate that direct coculture with epithelial-like feeder cells induces Ad-MCA formation in PDAC cells before the onset of EMT, and Ad-MCA formation converts GM-sensitive CD44v3-10^high^/CD44s^low^ PDAC cells into GM-resistant quiescent CSC-like cells. Furthermore, our work shows that the transcriptomes of PDAC cells are very rapidly and significantly changed by coculture with HEK293T cells. The rapid phenotypic changes of PDAC cells observed in this coculture system appear to mimic those occurring at the early phase of metastatic colonization of PDAC cells. This coculture system should be useful for understanding the molecular mechanisms underlying the emergence of intractable PDAC cells and the true nature of collective cell behavior.

## RESULTS

### Coculture with HEK293T cells induces Ad-MCA formation and GM resistance in epithelial cell phenotype CD44v^high^/CD44s^low^ PDAC cells

**Figure 1:**
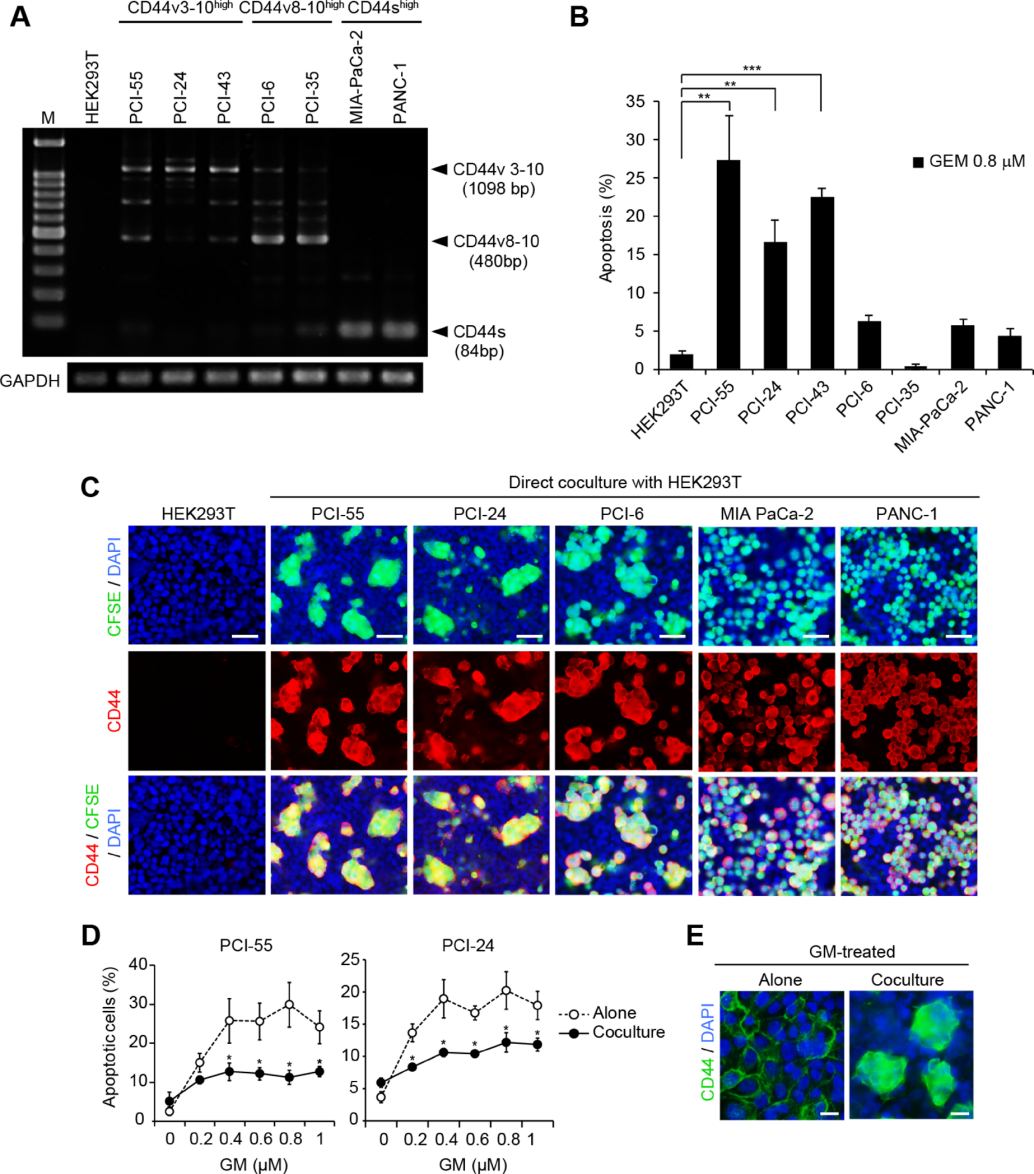
Direct coculture with HEK293T cells induces Ad-MCAs in CD44v^high^/CD44s^low^ epithelial PDAC cells (**A**) RT-PCR analysis of CD44 variant isoform expression in seven CD44+ PDAC cell lines. (**B**) Percentage of apoptotic PDAC cells induced by treatment with GM. PDAC cell lines were cultured in the presence of 0.8 μM GM for 48 h. Apoptotic PDAC cells were evaluated by the percentage of sub G0/G1 phase cells by flow cytometry. (**C**) Ad-MCA formation by CD44v^high^/CD44s^low^ epithelial PDAC cells. (**D**) Percentage of apoptotic cells in PCI-55 and PCI-24 cells treated with GM for 48 h. (**E**) Ad-MCA formation by PCI-55 cells is not affected by treatment with 0.8 μM GM (right). The data are presented as the mean values of three independent experiments. ^*^P < 0.05, ^**^P < 0.01, ^***^P < 0.001. Bars: 50 μm (C), 25 μm (E).

Altered expression of CD44 from CD44v to CD44s induces EMT and promotes cancer progression [10]. This suggests that the classification of splicing isoforms can be used as an indicator of the EMT process. Thus, to distinguish whether the PDAC cell lines used in this study exhibited an epithelial cell or mesenchymal cell phenotype, we examined the expression patterns of CD44 variant isoform transcripts in the following CD44+ PDAC cell lines: PCI-55, PCI-24, PCI-43, PCI-6, PCI-35, MIA-PaCa-2, and PANC-1 (Figure 1A). PCI-55, PCI-24, PCI-6, and PCI-35 cells showed an epithelial cell phenotype that exhibits high expression of CD44v mRNA and low expression of CD44s mRNA (CD44v^high^/CD44s^low^), of which PCI-55, PCI-24, and PCI-43 showed high expression of CD44v3-10 mRNA (CD44v3-10^high^/CD44s^low^), and PCI-6 and PCI-35 cells showed high expression of CD44v8-10 mRNA (CD44v8-10^high^/CD44s^low^). These CD44 variants were confirmed by direct sequencing of PCR products. In contrast, MIA-PaCa-2 and PANC-1 cells showed a mesenchymal cell phenotype, exhibiting low expression of CD44v mRNA and high expression of CD44s mRNA (CD44v^low^/CD44s^high^). Next, we evaluated GM sensitivity in each PDAC cell line by measuring the percentage of apoptotic cells induced by treatment with 0.8 μM GM for 48 h. PCI-55, PCI-24, and PCI-43 were more sensitive to GM (∼30% and ∼20% of apoptotic cells) than PCI-6, PCI-36, MIA-PaCa-2, and PANC-1 (less than 6% of apoptotic cells) (Figure 1B). Interestingly, PDAC cell lines expressing different CD44 isoforms showed different behavior when they were cocultured with HEK293T cells (Figure 1C). CD44v3-10^high^/CD44s^low^ PDAC cells such as PCI-55 and PCI-24, and CD44v8-10^high^/CD44s^low^ PDAC cells such as PCI-6 adhered to a monolayer of HEK293T cells and formed Ad-MCAs. In contrast, CD44v^low^/CD44s^high^ PDAC cells such as MIA-PaCa-2 and PANC-1 failed to form Ad-MCAs. We then examined whether coculture with HEK293T cells affected sensitivity to GM in GM-sensitive PCI-55 and PCI-24 cells. Coculture with HEK293T cells made PCI-55 and PCI-24 cells more resistant to GM (Figure 1D). Treatment with GM affected Ad-MCA formation by neither PCI-55 (Figure 1E) nor PCI-24 cells (data not shown). Taken together, these results indicate that coculture with HEK293T cells induces Ad-MCA formation and GM resistance in CD44v3-10^high^/CD44s^low^ PDAC cells.

### CD44v3-10^high^/CD44s^low^ PDAC cells forming Ad-MCAs upregulate CD44v8-10 expression

**Figure 2:**
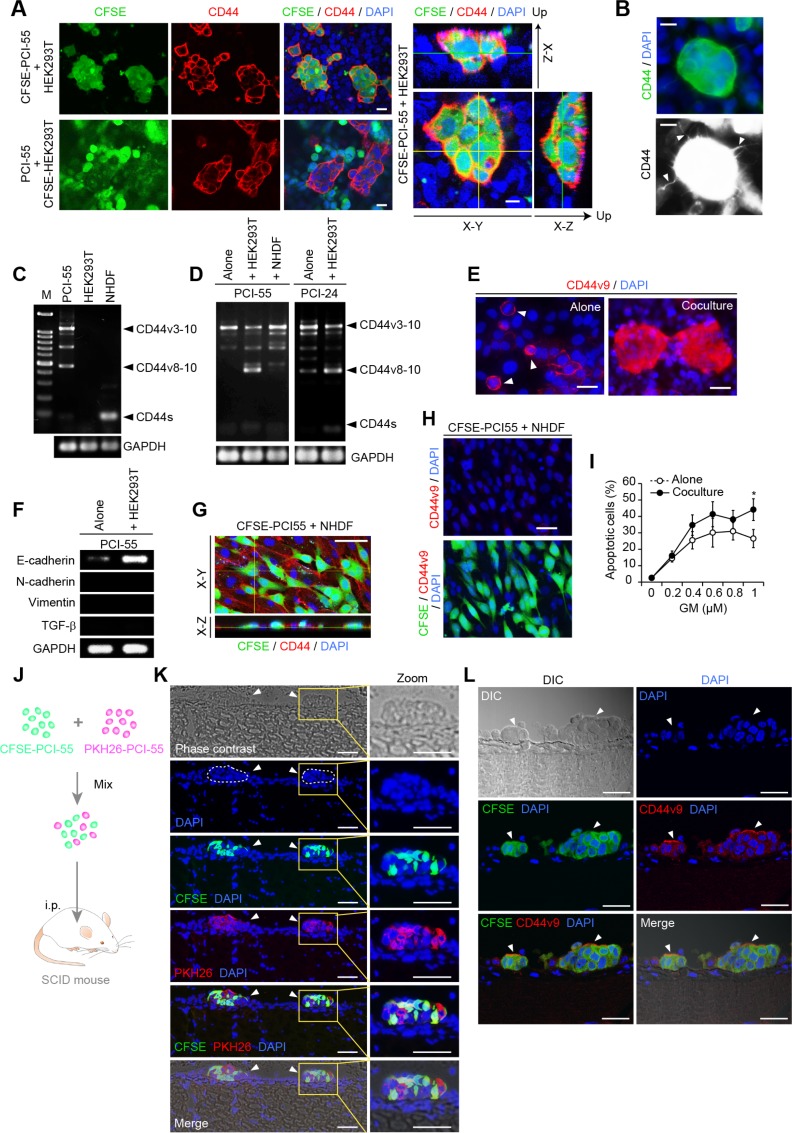
Ad-MCA-forming CD44v3-10^high^/CD44s^low^ PDAC cells increase CD44v8-10 expression (**A**) Pronounced staining for CD44 was observed on the surface of Ad-MCAs formed by PCI-55 cells on a monolayer of HEK293T cells. Trans-axial images (left panels) and three-dimensional images (right panels) were captured by confocal microscopy. (**B**) Filopodia on the surface of MCA. (**C**, **D**) RT-PCR analysis of CD44 variant isoform mRNAs. PDAC cells were sorted after culture without feeder cells or coculture with HEK293T or NHDFs. Alone (C) and coculture (D). (**E**) CD44v9 expression in PCI-55 cells cultured alone or cocultured with HEK293T cells. (**F**) RT-PCR analysis of expression of E-cadherin, N-cadherin, vimentin, and TGF-β. PDAC cells were sorted after culture without feeder cells or coculture with HEK293T cells. (**G**) Three-dimensional analysis of PCI-55 cells cocultured with NHDFs. (**H**) Absence of CD44v9 expression in CFSE-labeled PCI-55 cells cocultured with NHDFs. (**I**) Percentage of apoptotic PDAC cells cocultured with NHDFs in the presence of GM (right panels). The data are presented as the mean values of three independent experiments. ^*^P<0.05. Bars: 25 μm. (**J**–**L**) CD44s^low^ PDAC cells form Ad-MCAs through peritoneal dissemination in SCID mice. A mouse model for peritoneal dissemination (J). A mixture of CFSE- or PKH24-labeled PCI-55 cells (1 × 10^6^ in total) were injected intraperitoneally into SCID mice. Peritonea were harvested from the mice after 3 days. Spherical microtumors composed of a mixture of green and red fluorescent cells were frequently observed on peritoneum (K, arrowheads). Immunofluorescent staining for CD44v9 (L). Peritonea were harvested at day 1 after injection of CFSE-labeled PCI-55 cells. Arrowheads, microtumors by PCI-55 cells. Bars: 200 μm (K), 100 μm (L).

Trans-axial images of cocultured cells captured by confocal microscopy revealed that CD44 was expressed exclusively by Ad-MCA-forming PCI-55 cells (Figure 2A, left panels). Three-dimensional analysis showed strong and smooth membranous staining for CD44 on the surface of Ad-MCAs that anchored to a monolayer of HEK293T cells (Figure 2A, right panels). Immunofluorescence staining for CD44 revealed that filopodia were induced on the surface of some Ad-MCAs (Figure 2B). Next, we examined the expression of CD44 isoforms in sorted Ad-MCA-forming PDAC cells. HEK293T cultured alone did not express CD44 transcripts, and NHDFs cultured alone expressed only CD44s transcripts (Figure 2C). When PCI-55 and PCI-24 cells were cocultured with HEK293T cells, they markedly increased expression of CD44v8-10 (Figure 2D). Consistent with increased expression of CD44v8-10 transcripts, strong staining for CD44v9 was observed in Ad-MCA-forming PCI-55 cells cocultured with HEK293T cells (Figure 2F, lower panel) whereas PCI-55 cells cultured alone exhibited expression of CD44v9 only in mitotic cells (Figure 2F, arrowheads in upper panel). Additionally, PCI-55 cells cocultured with HEK293T cells increased expression of E-cadherin but not vimentin and transforming growth factor-β (TGF-β), suggesting that PCI-55 cells forming Ad-MCAs kept showing the epithelial cell phenotype (Figure 2E). Interestingly, unlike HEK293T cells, PCI-55 cells cocultured with NHDFs failed to form Ad-MCAs (Figure 2G). They neither increased expression of CD44v8-10 mRNA or CD44v9 protein (Figure 2H) nor acquired GM resistance (Figure 2I).

Next, we examined whether CD44v3-10^high^/CD44s^low^ PDAC cells form Ad-MCAs *in vivo* using a mouse model for peritoneal dissemination. To determine whether colonizing microtumors were formed by clonal proliferation or cellular aggregation of PCI-55 cells, a mixture of CFSE- or PKH26-labeled PCI-55 cells were injected intraperitoneally into SCID mice (Figure 2J). After 3 days, spherical microtumors composed of a mixture of green- and red-fluorescent cells were observed frequently on peritonea, indicating that PCI-55 cells formed Ad-MCAs (Figure 2K, arrowheads). Next, to perform immunofluorescent staining for CD44v9, peritonea were harvested from SCID mice at day 1 after injection of CFSE-labeled PCI-55 cells. Positive staining for CD44v9 was observed on the surface of Ad-MCAs by PCI-55 cells (Figure 2L, arrowheads). These results indicate that PCI-55 cells form Ad-MCAs on peritoneum through peritoneal dissemination *in vivo* and show expression of CD44v9.

### CD44v8-10^high^/CD44s^low^ PDAC cells forming Ad-MCAs are in a quiescent state

**Figure 3:**
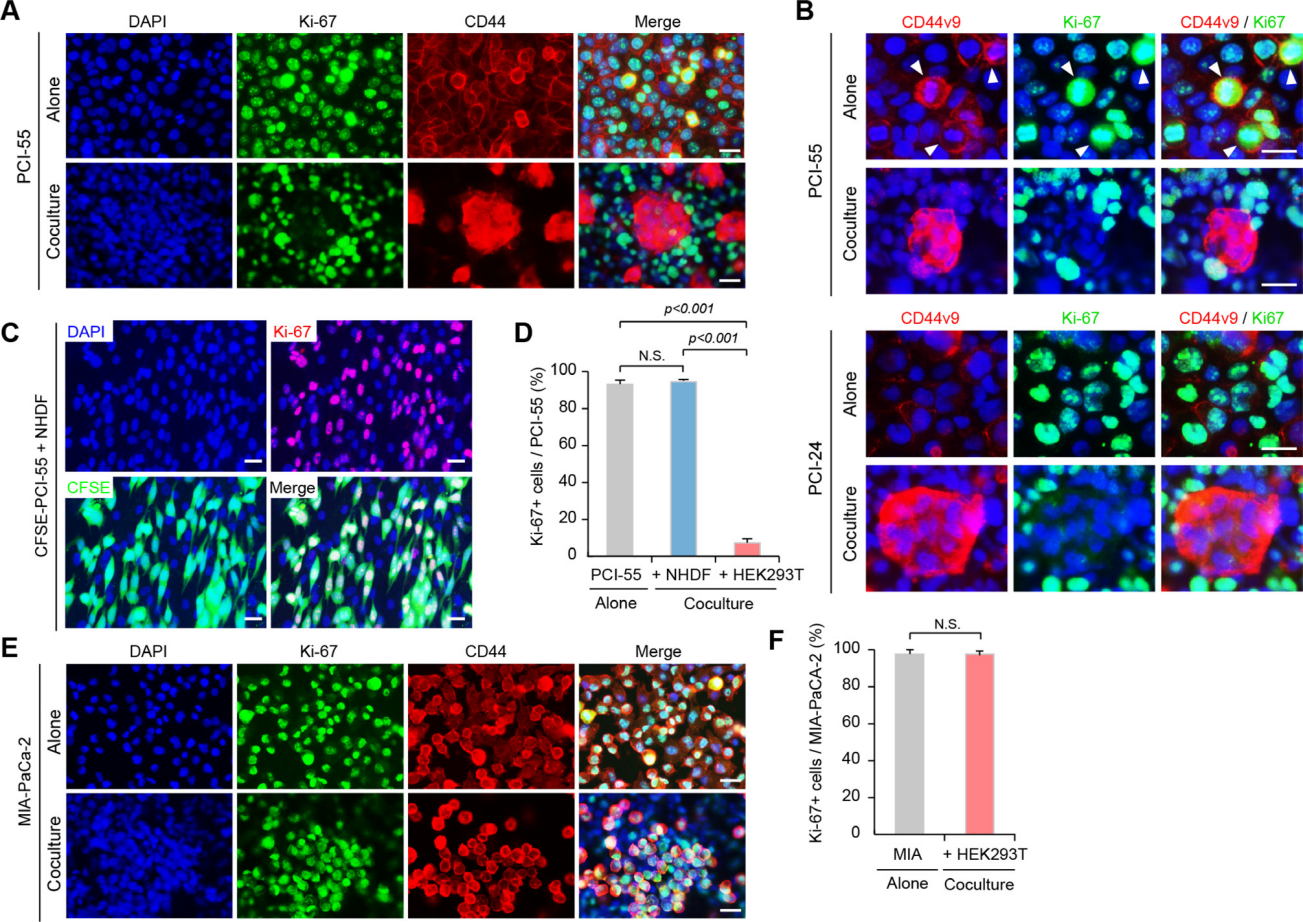
Ad-MCA-forming CD44v8-10^high^/CD44s^low^ PDAC cells enter a quiescent state (**A**) Expression of Ki-67 and CD44 in PCI-55 cells cocultured with HEK293T cells overnight. (**B**) Expression of Ki-67 and CD44v9 in PCI-55 and PCI-24 cells. (**C**) Expression of Ki-67 in PCI-55 cells cocultured with NHDFs overnight. (**D**) Percentage of Ki-67+ cells in PCI-55 cells cultured alone (left), cocultured with NHDFs (middle), and cocultured with HEK293T (right). (**E**) Expression of Ki-67 and CD44v9 in MIA-PaCa-2 cells. (**F**) Percentage of Ki-67+ cells in MIA-PaCa-2 cells cultured alone (left) and cocultured with HEK293T (right). NS, not significant. Positive cells were counted in 20 randomly selected high-power fields (×200). Bars: 25 μm.

After coculture with HEK293T cells, CD44v3-10^high^/CD44s^low^ PDAC cells markedly increased CD44v8-10 expression and resistance to GM (Figure 2), suggesting that they had acquired CSC-like more malignant properties. To examine whether Ad-MCA-forming PDAC cells are in a quiescent state, we performed immunofluorescence staining for Ki-67, a nuclear protein expressed during all active phases of the cell cycle (G1, S, G2, and M), but absent in resting G0 phase [23]. When PCI-55 cells were cultured alone, the majority of cells exhibited positive nuclear staining for Ki-67 (Figure 3A, upper panels). However, when PCI-55 cells were cocultured with HEK293T cells, positive staining was dramatically diminished in MCA-forming cells (Figure 3A, lower panels). Interestingly, positive staining for CD44v9 was observed in Ki-67-negative, MCA-forming PCI-55 cells; however, when cultured alone, positive staining for CD44v9 was observed in Ki-67-positive PCI-55 cells (Figure 3B, upper panels). Similar staining patterns were observed in PCI-24 cells (Figure 3B, lower panels). In sharp contrast, the majority of PCI-55 cells cocultured with NHDFs retained positive staining for Ki-67 (Figure 3C). Ki-67-positive cells occupied 93% of PCI-55 cells cultured alone, 94% of PCI-55 cells cocultured with NHDFs, and only 7% of PCI-55 cells forming Ad-MCAs (Figure 3D). In addition, most MIA-PaCa-2 cells retained positive staining for Ki-67 even when they were cocultured with HEK293T cells (Figure 3E and 3F). Similar results were observed in PANC-1 cells (data not shown). These results indicate that PCI-55 and PCI-24 cells form Ad-MCAs and enter a quiescent state when cocultured with HEK293T cells, but not with NHDFs.

### Transcriptomes of PDAC cells were rapidly and significantly changed by coculture with HEK293T cells

**Figure 4:**
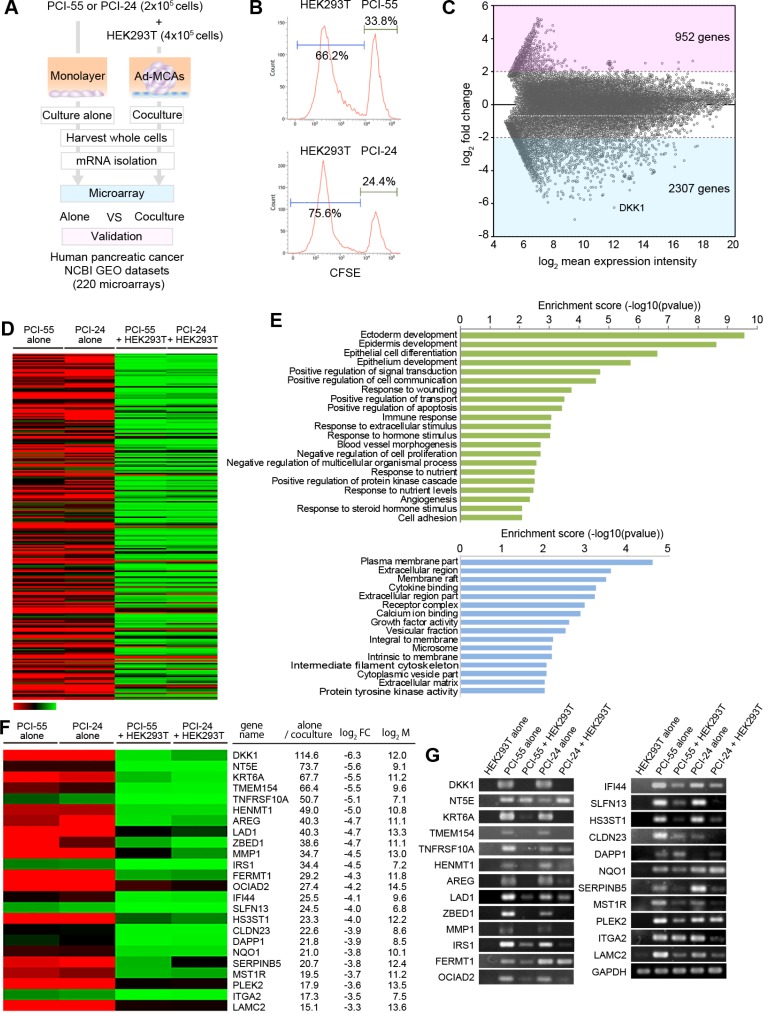
Comparison of transcriptomes between PDAC cells cultured alone and PDAC cells cocultured with HEK293T cells (**A**) Flow chart for the microarray analysis. (**B**) The ratio of PDAC cells and HEK293T cells in coculture samples (%). CSFE-labeled PDAC cells were co-cultured with a monolayer of HEK293T cells. The ratio was examined by FCM analysis. (**C**) M-A plot showing fold-change in transcript expression between PDAC cells alone and PDAC cells cocultured with HEK293T cells according to the mean expression intensity. We employed the mean value of the expression levels in two PDAC cell lines for each gene, which is represented as a dot. (**D**) Heat-map showing transcriptomes in PDAC cells cultured alone (*n* = 2) vs. PDAC cells cocultured with HEK293T cells (*n* = 2). (**E**) Gene Oncology (GO) analysis of genes downregulated following coculture with HEK293T cells (<0.1). GO terms belonging to biological processes (BP) are shown in green. Molecular functions (MF) and cellular components (CC) are shown in blue. GO terms were sorted based on *P*-values. Genes with ≤ 0.1 fold-change and *P* < 0.05 were selected as differentially expressed genes (DEGs). (**F**) Heat-map of some DEGs as determined by significance analysis of microarray. (**G**) Differentially expressed genes were validated by RT-PCR.

To examine the transcriptomes in PDAC cells forming Ad-MCAs, we performed microarray analysis using total RNA isolated from PDAC cells cocultured alone and PDAC cells cocultured with HEK293T cells (Figure 4A). To protect PDAC cells forming Ad-MCAs and avoid secondary changes to their gene profiles by cell sorting, we used whole cell lysates containing PDAC cells and HEK293T cells. To examine the ratio of PDAC cells and 293T cells, we performed flow cytometry analysis using CFSE-labeled PDAC cells cocultured with unlabeled HEK293T cells overnight prior to performing microarrays. The contents of PCI-55 cells and PCI-24 cells were 33.8% and 24.4%, respectively (Figure 4B). When we compared transcriptomes between PDAC cells cultured alone and PDAC cells cocultured with HEK293T cells, 2,307 genes were significantly downregulated by coculture with HEK293T cells whereas 952 genes were upregulated (Figure 4C). To avoid contamination of the transcriptome of HEK293T cells, we focused on genes that were largely downregulated (≤0.1 fold-change) by coculturing with HEK293T cells (Figure 4D). To determine the enrichment of genes and gene products in biological processes (BP), molecular functions (MF), and cellular components (CC), we performed Gene Oncology (GO) analysis of genes with ≤0.1 fold-change and *P* < 0.05 by coculture with HEK293T cells (Figure 4E). GO analysis of BP showed that the most prominently downregulated gene set by coculture with HEK293T cells is involved in ectoderm development (Figure 4E, upper panel). GO analysis of MF and CC showed that downregulated gene sets by coculture are involved in the plasma membrane part of the extracellular region (Figure 4E, lower panel).

### Twenty-four genes highly downregulated in PDAC cells cocultured with HEK293T cells are upregulated in malignant lesions from pancreatic cancer patients

**Figure 5:**
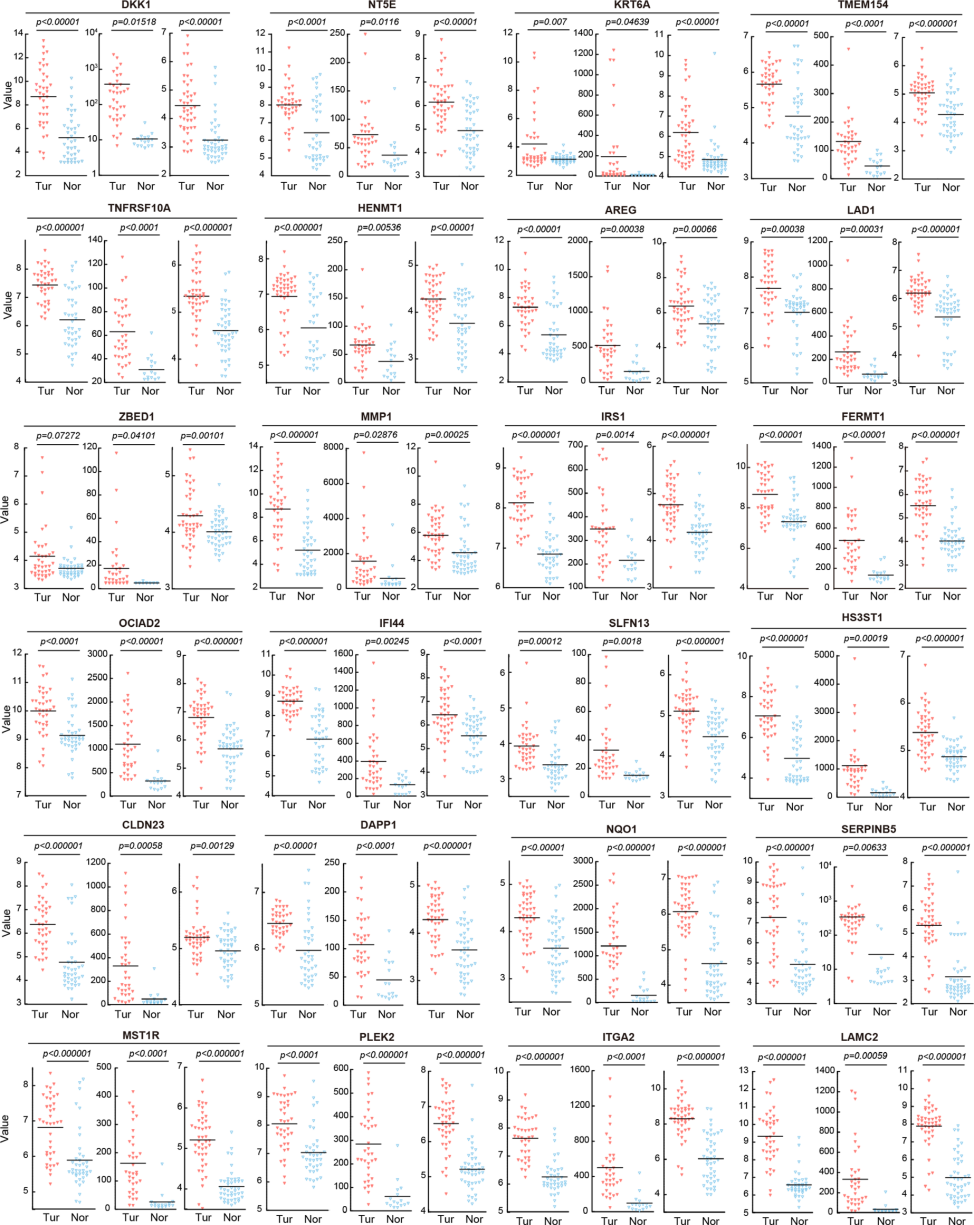
Validation of 24 DEGs by three microarray datasets in NCBI Gene Expression Omnibus (GEO) Twenty-four DEGs down-regulated by coculture with HEK293T cells were upregulated in the lesions of malignant tumors resected from pancreatic cancer patients compared with normal adjacent tissue. Three independent pancreatic cancer datasets obtained from surgically excised tissues were curated from GEO, with a total of 220 microarrays. GDS4103 (left graph), GDS4102 (middle graph), and GDS4336 (right graph). GDS4103 (ICF cohort: whole-tissue PDAC): analysis of PDAC tumors and matching normal pancreatic tissue from pancreatic cancer patients, totaling 72 samples (tumors *n* = 32, non-tumors *n* = 32). GDS4102 (pancreatic tumor and normal tissue samples): analysis of tumor tissue and normal tissue in pancreatic cancer samples, totaling 52 samples (tumors *n* = 32, non-tumors *n* = 16). GDS4336 (PDAC tumor and adjacent non-tumor tissue tumors): analysis of 45 matching pairs of PDAC tumor and adjacent non-tumor tissue, totaling 90 samples (tumors *n* = 45, non-tumors *n* = 45). Tur, tumors; Nor, non-tumors.

Finally, to validate the clinical relevancy of the gene profile obtained from PDAC cells cocultured with HEK293T cells, we employed three data sets from pancreatic cancer patients, which were downloaded from publicly available microarray data on NCBI Gene Expression Omnibus (GEO), and compared with genes downregulated by the coculture. Specifically, three independent pancreatic cancer datasets obtained from surgically excised tissues were curated from GEO. These data sets consist of a total of 220 microarrays: GDS4103 (tumors *n* = 32, non-tumors *n* = 32, left dot plot), GDS4102 (tumors *n* = 32, non-tumors *n* = 16, middle dot plot), and GDS4336 (tumors *n* = 45, non-tumors *n* = 45, right dot plot). Interestingly, we found that genes downregulated in PDAC cells cocultured with HEK293T cells were frequently upregulated in malignant lesions resected from pancreatic cancer patients when compared with their normal adjacent tissue (Figure 4F, 4G and Figure 5). We identified 24 differentially expressed genes (DEGs) in PDACs that were significantly downregulated by PDAC cells cocultured with HEK293T cells: dickkopf-related protein 1 (*DKK1*), 5′-nucleotidase (*NT5E*), keratin 6A (*KRT6A*), transmembrane protein 154 (*TMEM154*), tumor necrosis factor receptor superfamily member 10A (*TNFRSF10A*), HEN methyltransferase 1 (*HENMT1*), amphiregulin (*AREG*), ladinin-1 (*LAD1*), zinc finger BED domain-containing protein 1 (*ZBED1*), matrix metalloproteinase-1 (*MMP1*), insulin receptor substrate 1 (*IRS1*), fermitin family member 1 (*FERMT1*), ovarian carcinoma immunoreactive antigen-like protein (*OCIAD2*), interferon-induced protein 44 (*IFI44*), schlafen family member 13 (*SLFN13*), heparan sulfate glucosamine 3-O-sulfotransferase 1 (*HS3ST1*), claudin 23 (*CLDN23*), dual adaptor of phosphotyrosine and 3-phosphoinositides 1 (*DAPP1*), NAD(P)H quinone dehydrogenase 1 (*NQO1*), serpin family B member 5 (*SERPINB5*), macrophage stimulating 1 receptor (*MST1R*), pleckstrin-2 (*PLEK2*), integrin alpha-2 (*ITGA2*), and laminin subunit gamma 2 (*LAMC2*) (Figure 4F). These gene expression patterns were validated by RT-PCR (Figure 4G). To clarify the gene profile of PDAC cells cocultured with HEK293T cells, we attempted to separate PDAC cells from HEK293T cells by cell sorting. However, many DEGs in PDAC cells cocultured with HEK293T cells were secondarily affected by cell sorting with disruption of Ad-MCA formation (data not shown). This result suggests that gene profiles in PDAC cells may be rapidly changed by Ad-MCA formation, and PDAC cells forming Ad-MCAs partially revert back to the gene profiles observed in normal tissue.

## DISCUSSION

We have demonstrated here that overnight coculture with HEK293T cells induces Ad-MCA formation in GM-sensitive, CD44v3-10^high^/CD44s^low^ PDAC cells, and that MCA-forming PDAC cells undergo phenotypic conversion into GM-resistant, CD44v8-10+, quiescent CSC-like PDAC cells (Figures 1 and 2). This coculture system provides a simple, convenient, and highly efficient means to obtain CSC-like refractory PDAC cells by inducing collective cell behavior like Ad-MCA formation. Feeder cells have been used extensively to support the growth and stemness potential of normal stem cells in *in vitro* cultures [24]. Our present study shows that feeder cells are also important for inducing phenotypic conversion in the population of cancer cells and presumably for supporting their stem-like phenotypes. HEK293T cells, which formed a coherent monolayer reminiscent of epithelial cells, promoted the phenotypic conversion of CD44v3-10^high^/CD44s^low^ cells to quiescent CSC-like cells, but NHDFs lacked such capacity (Figure 3). This observation, which underscores the importance of ‘appropriate’ feeder cells, might to some degree reflect the importance of the microenvironment for inducing and supporting CSCs *in vivo*.

Another notable observation is that phenotypic conversion to PDAC cells with CSC-like intractable phenotypes occurred only in CD44v3-10^high^/CD44s^low^ PDAC cells such as PCI-55 and PCI-24 (Figures 1 and 2). Current evidence indicates that CD44s+ cancer cells displaying EMT phenotypes are more intractable than those expressing other CD44 isoforms [10]. In breast cancer, a shift in CD44 expression from CD44v to CD44s occurs during EMT, and this isoform switching is essential for cells to undergo EMT [10]. CD44s^high^ GM-resistant PDAC cells such as MIA-PaCa-2 and PANC-1 lacked the ability to form solid Ad-MCAs when cocultured with HEK293T cells and administered to NOD-SCID mice intraperitoneally *in vivo* (data not shown). This might be because they have acquired a mesenchymal phenotype. On the other hand, PCI-6 cells were GM-resistant, but still kept the ability to form Ad-MCAs and markedly increased CD44s expression when cocultured with HEK293T cells. These observations suggest that PCI-6 cells cultured on HEK293T feeder cells might be poised to undergo EMT. Coculture with HEK293T cells induced CD44v8-10 expression and cellular quiescence in CD44v3-10^high^/CD44s^low^ PDAC cells (Figure 3), but was not able to appreciably induce isoform switching to CD44s. Thus, for CD44v3-10^high^/CD44s^low^ PDAC cells, coculture with HEK293T alone appears insufficient to induce a mesenchymal phenotype. Based on the behaviors of the PDAC cell lines discussed above, it is tempting to speculate that PDAC cells increase their malignant potential from CD44v3-10^high^/CD44s^low^ cells to CD44v8-10^high^/CD44s^low^ CSC-like cells, and then to mesenchymal CD44v^low^/CD44s^high^ cells with high metastatic colonization potential such as peritoneal dissemination.

By using three data sets in GEO, we found that 24 highly downregulated genes in cocultured PDAC cells were upregulated in malignant lesions surgically excised from pancreatic cancer patients (Figures 4 and 5). These results indicate that the CSC-like refractory PDAC cells forming Ad-MCAs partially return to their normal tissue gene profile. Our results also suggest that the transcriptome of PDAC primary tumors can be greatly altered by forming Ad-MCAs at the early stages of metastatic colonization such as peritoneal dissemination *in vivo*. Thus, when seeking for candidates for prognostic biomarkers and therapeutic targets, it is necessary to consider that there may be significant differences between the gene profiles of PDAC primary tumors and metastatic colony-forming PDAC tumors.

In conclusion, we have developed a direct coculture system that efficiently converts CD44v3-10^high^/CD44s^low^ PDAC cells into quiescent CD44v8-10^high^ CSC-like intractable PDAC cells by forming Ad-MCAs. HEK293T cells used as feeders for inducing the collective cell behavior are easy to grow and widely used in cell biology research. The coculture system described here should be useful for elucidating the true nature of collective cell behavior of PDAC cells and also for developing new therapeutic reagents against the refractory cancer. This coculture system is likely also useful for understanding the molecular mechanisms underlying the emergence of Ad-MCAs by the population of PDAC cells that are out of mitosis and may mimic *in vivo* events occurring at the very early stages of metastatic colonization such as peritoneal dissemination.

## MATERIALS AND METHODS

### Cells

Seven human PDAC lines, PCI-55, PCI-24, PCI-43, PCI-6, PCI-35, MIA-PaCa-2, and PANC-1, were used. PCI-55, PCI-24, PCI-43, PCI-6, and PCI-35 were established from pancreatic carcinoma tissues of primary sites surgically resected at Hokkaido University Hospital [25]. HEK293T cells, human embryo kidney-derived epithelial-like cells, were used as normal tissue-derived feeder cells. PDAC cell lines and HEK293T cells were maintained in Dulbecco's minimal essential medium (DMEM) supplemented with 10% fetal bovine serum (FBS) and penicillin/streptomycin. Primary normal human dermal fibroblasts (NHDF) obtained from TaKaRa Biomedicals (Shiga, Japan) were used as normal tissue-derived mesenchymal cells.

### Antibodies and reagents

Rat anti-human/mouse CD44 (clone IM7; eBioscience Inc., San Diego, CA), rat anti-human CD44v9 (clone RV3; CosmoBio, Tokyo, Japan), and mouse anti-human Ki-67 (clone MIB-1; Dako, Glostrup, Denmark) antibodies were used for immunofluorescence staining. GM was purchased from Sigma-Aldrich (St. Louis, MO). 5- (and 6-) carboxyfluorescein diacetate succinimidyl ester (CFSE; Dojindo laboratories, Kumamoto, Japan), and PKH26 (Sigma-Aldrich, St. Louis, MO) were used for live-cell labeling.

### Coculture

PDAC cells were cocultured with HEK293T cells or NHDFs using a direct coculture system that allowed for cell-cell contact. PDAC cells or feeder cells were labeled with 1 μmol/L of CFSE. A total of 2 × 10^5^ PDAC cells were directly cocultured with feeder cells growing as monolayers in 24-well plates.

### Apoptosis assay

Apoptotic PDAC cells were evaluated by the percentage of sub G0/G1 phase cells in CFSE-negative cells using the propidium iodide staining method, as previously described [18]. Analysis was performed using CellQuest Pro software version 6.0 (Becton Dickinson Immunocytometry Systems, San Jose, CA).

### Flow cytometry sorting

PDAC cells cocultured with CFSE-labeled HEK293T cells overnight were trypsinized and washed in PBS. Cocultured cells were resuspended at a concentration of 5 × 10^6^ cells/mL in PBS supplemented with 3% FBS. Cells were then analyzed and sorted by FACSAria (Becton Dickinson, Franklin Lakes, NJ).

### Immunofluorescence staining

This was performed as previously described [18]. Images were acquired using an Olympus DP70 camera with its own Olympus DP controller software (Olympus, Tokyo, Japan). Three-dimensional analysis was conducted using images acquired by a laser scanning confocal microscope (FV-1000D; Olympus, Tokyo, Japan).

### Mice

C.B-17/lcr-scid/scidJcl mice were purchased from CLEA Japan, Inc. (Tokyo, Japan). Mice were bred and housed under specific pathogen-free conditions in our animal facility. All of the animal experiments were conducted according to the Guidelines for the Care and Use of Laboratory Animals at Hokkaido University Faculty of Medicine and approved by the Institutional Review Committee of Hokkaido University.

### Mouse model for peritoneal dissemination

CFSE- or PKH26-labeled PCI-55 cells (5 × 10^6^ cells in total) were suspended in 200 μl of PBS and injected intraperitoneally into 6- to 8-week-old male mice with severe combined immunodeficiency (SCID). Peritonea were harvested at indicated times after injection and prepared for frozen sections with Tissue-Tek O.C.T. Compound (Sakura Finetek Japan, Tokyo, Japan).

### Microarray analysis

Total RNAs were isolated from whole PDAC cells cultured alone and PDAC cells cocultured with HEK293T cells. Cyanine-3 (Cy3) labeled cRNA was prepared from 0.1 μg total RNA using the Low Input Quick Amp Labeling Kit (Agilent Technologies, Santa Clara, CA, USA) according to the manufacturer's instructions. The labeled cRNAs were hybridized onto the microarray. After washing, the arrays were scanned by the Agilent SureScan Microarray Scanner (G2600D). The scanned images were analyzed with Feature Extraction Software 11.5.1.1 (Agilent) using default parameters to obtain background subtracted and spatially detrended processed signal intensities.

### Statistical analysis

Data were analyzed using the Student's *t*-test. *P* values less than 0.05 were considered significant.
